# Lipid, Detergent, and Coomassie Blue G-250 Affect the Migration of Small Membrane Proteins in Blue Native Gels: MITOCHONDRIAL CARRIERS MIGRATE AS MONOMERS NOT DIMERS*

**DOI:** 10.1074/jbc.M113.484329

**Published:** 2013-06-06

**Authors:** Paul G. Crichton, Marilyn Harding, Jonathan J. Ruprecht, Yang Lee, Edmund R. S. Kunji

**Affiliations:** From the Mitochondrial Biology Unit, Medical Research Council, Hills Road, Cambridge CB2 0XY, United Kingdom

**Keywords:** Gel Electrophoresis, Membrane Proteins, Mitochondrial Transport, Protein Aggregation, Transporters, Detergent Micelle

## Abstract

Blue native gel electrophoresis is a popular method for the determination of the oligomeric state of membrane proteins. Studies using this technique have reported that mitochondrial carriers are dimeric (composed of two ∼32-kDa monomers) and, in some cases, can form physiologically relevant associations with other proteins. Here, we have scrutinized the behavior of the yeast mitochondrial ADP/ATP carrier AAC3 in blue native gels. We find that the apparent mass of AAC3 varies in a detergent- and lipid-dependent manner (from ∼60 to ∼130 kDa) that is not related to changes in the oligomeric state of the protein, but reflects differences in the associated detergent-lipid micelle and Coomassie Blue G-250 used in this technique. Higher oligomeric state species are only observed under less favorable solubilization conditions, consistent with aggregation of the protein. Calibration with an artificial covalent AAC3 dimer indicates that the mass observed for solubilized AAC3 and other mitochondrial carriers corresponds to a monomer. Size exclusion chromatography of purified AAC3 in dodecyl maltoside under blue native gel-like conditions shows that the mass of the monomer is ∼120 kDa, but appears smaller on gels (∼60 kDa) due to the unusually high amount of bound negatively charged dye, which increases the electrophoretic mobility of the protein-detergent-dye micelle complex. Our results show that bound lipid, detergent, and Coomassie stain alter the behavior of mitochondrial carriers on gels, which is likely to be true for other small membrane proteins where the associated lipid-detergent micelle is large when compared with the mass of the protein.

## Teaser

**Background:** Mitochondrial carriers were thought to be dimeric based on their migration in blue native gels.

**Results:** The high molecular mass species observed in blue native gels are composed of protein monomers, detergent, lipid, and Coomassie stain.

**Conclusion:** The mitochondrial carriers are monomeric not dimeric.

**Significance:** The apparent mass of small membrane proteins in blue native gels requires significant correction.

## Introduction

Mitochondrial transport proteins or carriers facilitate the exchange of various metabolites across the mitochondrial inner membrane, including nucleotides, vitamins, keto and amino acids, and inorganic ions. These transport steps are essential for many biochemical processes, such as oxidative phosphorylation, the synthesis of iron sulfur clusters and heme, and the synthesis of DNA and RNA as well as the synthesis, degradation, and interconversion of amino acids (1). Several carriers are associated with genetic disorders (2), whereas uncoupling proteins (*e.g.* UCP1), which also belong to the transporter family, may be important in our understanding and treatment of obesity (3).

Mitochondrial carriers share the same basic structure (4) and are likely to operate by a common mechanism (5). The structural fold consists of three ∼100-amino acid repeat domains that each form two trans-membrane α-helices separated by a matrix loop and small matrix α-helix (4, 6, 7). The first helix of each domain contains a signature motif P*X*[DE]*XX*[RK], which is well conserved across all the members of this protein family (6).

For over 30 years, mitochondrial carriers were believed to be homodimers, composed of two ∼32-kDa proteins. Early experiments indicated that inhibitors bound to carriers in a 1:2 stoichiometry (8–10). Since then, observations made using many techniques and experimental approaches, predominantly with the ADP/ATP carrier and UCP1, were consistent with a structural dimer. Gel filtration (11, 12), equilibrium sedimentation (11, 12), small-angle neutron scattering (13), differential affinity purification (14), and native gel electrophoresis (15–21) studies all indicated that carriers were dimeric in detergent, whereas freeze-fracture electron microscopy (22) and chemical cross-linking (23, 24) experiments indicated that carriers were dimeric in lipid membranes. Furthermore, the kinetics of transport (see Ref. 25 for overview), reconstitution into liposomes, and negative dominance (14) could be explained with a dimer model of carrier function.

A dimer arrangement of carriers was challenged when the first structural information was obtained for the ADP/ATP carrier in complex with the inhibitor carboxyatractyloside (CATR).^2^ Both the projection map of the yeast protein (26) and the atomic structure of the bovine protein (4) revealed a monomeric fold. The six trans-membrane α-helices from the three repeat domains form a barrel arrangement with three-fold pseudo-symmetry, where the P*X*[DE]*XX*[RK] motif from each domain is able to form a salt bridge network that closes a central cavity on the matrix side (4). Further investigations with the yeast ADP/ATP carriers in gel filtration (27), analytical ultracentrifugation (27), differential affinity purification (28), and negative dominance studies (29) indicated the presence of only monomers. Similarly, the bovine isoform was reinvestigated in analytical ultracentrifugation and small angle neutron scattering experiments and found to be predominantly monomeric (30), in contrast to past conclusions. A re-evaluation of much of the earlier work has shown that with the benefit of hindsight, most of the observations are consistent with the carriers being monomeric (see Ref. 31 for review). Confirming the oligomeric status of mitochondrial carriers is crucial for understanding the transport mechanism.

Blue native-PAGE is a relatively inexpensive and convenient sizing technique to study proteins in nondenaturing conditions. As such, it has been used in many studies to assess the oligomeric state of mitochondrial carriers. In a similar manner to SDS-PAGE, proteins are separated by size, but the milder Coomassie dye is used instead of SDS to provide the necessary negative charge for the electrophoretic separation of proteins under native conditions. Mitochondrial carriers are reported to migrate on gels with molecular masses between 65 and 120 kDa (15–21), which have been interpreted to be homodimers. Higher mass species have also been observed and were thought to be associations with other proteins (32, 33). More recently, the migration of the ADP/ATP carrier, along with other membrane proteins, was used to validate migration-mass relationships on blue native gels and was believed to represent a dimer (∼66 kDa) (34). The observations from blue native-PAGE studies are inconsistent with recent data obtained using various other techniques (27–30).

Here, we have investigated the behavior of the yeast ADP/ATP carrier (AAC3) on blue native gels. We find that both lipids and detergent vary the apparent mass of AAC3, which is not related to changes in the oligomeric state of the protein. When the effects are minimalized, AAC3 migrates with an apparent molecular mass of ∼60 kDa, yet is monomeric, as confirmed by controls using genetically fused dimers. The protein binds an unusually high amount of Coomassie dye when compared with other membrane proteins, which can explain the migration pattern observed in blue native gels.

## MATERIALS AND METHODS

### 

#### 

##### Cloning of Yeast Expression Vectors and Transformation

The yeast *aac3* gene was cloned into the pYES-P*aac2-aac2* vector replacing *aac2*, resulting in the expression vector pYES-P*aac2-aac3*, and an N-terminal nine-histidine tag and a factor Xa cleavage site were introduced at the NcoI site by kinase treatment and annealing of synthesized primers, leading to the vector pYES-P*aac2*-N9His-Xa-*aac3* (26). For the construction of the expression vector for the covalently linked AAC3 dimer, the stop codon of the first *aac3* gene was replaced by an XhoI restriction site and the start codon of the second *aac3* gene was replaced by an XhoI site by PCR. The two DNA fragments were cloned in tandem behind the P*aac2* promotor using the common XhoI site, yielding a tandem construct with a Leu-Glu linker. All cloned vectors were isolated by miniprep (Qiagen) and confirmed by PCR, restriction analysis, and sequencing (Cambridge Bioscience). *Saccharomyces cerevisiae* strain WB-12 (MATα *ade2-1 trp1-1 ura3-1 can1-100 aac1*::*LEU2 aac2*::*HIS3*), lacking functional AAC1 and AAC2 carriers, was a gift from Dr. H. Terada (35). The plasmids were transformed into the WB-12 strain using standard techniques, and transformants were selected on SC medium −Trp plates (Invitrogen).

##### Preparation of Mitochondrial Membranes

Yeast (strain WB12) expressing functional AAC3 or covalently linked AAC3 dimer were grown aerobically in 3% glycerol YPG medium (4 × 500 ml in 2-liter flasks) at 30 °C to an *A*_600_ of ∼5. Mitochondrial membranes were isolated from 20 to 30 g of wet weight of cells using a cell disruptor as described previously (28). Aliquots of mitochondrial membranes (∼10 mg/ml protein) were flash-frozen and stored at −80 °C until further use. For His-tagged AAC3 purification, yeast were grown in a 55-liter fermentor, and mitochondrial membranes were isolated as described in Ref. 27.

Brown adipose tissue was isolated from newborn lambs that had died of natural causes (University of Cambridge Veterinary School) and was stored in liquid nitrogen. Mitochondria were isolated using established methods (36) and stored in liquid nitrogen.

##### Protein Purification

His-tagged AAC3 was purified by nickel affinity chromatography based on a procedure described previously (27). Approximately 500 mg of yeast mitochondrial membranes was thawed from storage and incubated with 2 ng of CATR (Sigma-Aldrich) per mg of mitochondrial protein for 20 min with mixing at 4 °C. Membranes were solubilized in a 2% undecyl-β-d-maltoside (11M) solution for 30 min at 4 °C containing 150 mm NaCl, 20 mm imidazole, 10 mm Tris, pH 7.4, and two tablets of Complete protease inhibitor minus EDTA per 100 ml (Roche Diagnostics). Insoluble material was removed by centrifugation (140,000 × *g* for 20 min, 4 °C), and the supernatant was loaded onto a nickel-Sepharose column (high performance; GE Healthcare) at 1 ml/min using an ÄKTAprime FPLC system. The column was washed at 3 ml min^−1^ with 100 ml of buffer A (containing 150 mm NaCl, 60 mm imidazole, 10 mm Tris, pH 7.4, with 0.2% decyl-β-d-maltoside (10M), 0.2% 11M, 0.1% dodecyl-β-d-maltoside (12M), or 0.1% tridecyl-β-d-maltoside (13M) included) followed by 30 ml of buffer B (containing 50 mm NaCl, 10 mm Tris, pH 7.4, and the same detergent as in buffer A). To cleave the protein from the column, the nickel-Sepharose was recovered as a slurry (∼1.2 ml) and treated with factor Xa protease overnight at 10 °C (60 units with 5 mm CaCl_2_ added; New England Biolabs Ltd.). The slurry was transferred to an empty micro bio-spin column (Bio-Rad Laboratories) and centrifuged (500 × *g*, 5 min at 4 °C) to elute the protein from the resin. Residual nickel-Sepharose contamination was pelleted by further centrifugation (12,000 × *g*, 10 min at 4 °C) in a 2-ml tube, and the purified AAC3 protein (0.5–1.3 mg/ml) was recovered in the supernatant. The final sample was quantified by BCA protein assay (Thermo Scientific) with bovine serum albumin as a standard.

##### Lipid Extraction

Mitochondrial lipids were extracted using a methanol/chloroform procedure adapted from Ref. 37. Yeast mitochondrial membranes (100 mg of protein) were diluted to 5 ml with distilled water and shaken vigorously with 18.8 ml of methanol:chloroform (2:1) in a glass-stoppered tube for 10 min. This was supplemented with 6.3 ml of chloroform and a few grains of butylated hydroxytoluene, shaken for 1 min, and supplemented further with 6.3 ml of distilled water and shaken again. The mixture was centrifuged at 4000 × *g* using a swing-out rotor, and the resulting protein disc between the aqueous and organic phases was carefully removed and homogenized in 6.25 ml of chloroform. The homogenate was added back to the biphasic system, and the mixture was recentrifuged. The lower organic phase containing the lipids was recovered and dried down under nitrogen, and the resulting lipid smear was redissolved in 2 ml of diethyl ether and redried. The lipid was mixed with 5 ml of 2% 12M under a nitrogen stream and stirred at 4 °C overnight. This sample was assumed to contain total mitochondrial lipid and was mixed with AAC3 and detergent accordingly to achieve the desired fraction of lipids and detergent present in the equivalent mitochondrial samples loaded on to gels.

##### Electrophoresis

Blue native PAGE was performed using established protocols (38). 5–13% or 6–18% (w/v) polyacrylamide linear gradient gels with a 4% (w/v) stacking gel were made using a gradient mixer apparatus and a conventional electrophoresis unit (SE 260 Series Mighty Small II) set up in a cold room. The light and heavy acrylamide solutions, containing 0.5 m aminohexanoic acid, 25 mm imidazole/HCl, pH 7.0, and 10% (w/v) glycerol (heavy solution only), were mixed and cast as described (38). The gel dimensions were 10 cm × 8 cm × 1.5 mm.

Mitochondrial membrane aliquots (∼500 μg of protein) were thawed from −80 °C storage, suspended in 50 μl of sample buffer (50 mm imidazole/HCl, pH 7, 50 mm NaCl, 5 mm 6-amino-hexanoic acid) and, in most cases, treated with 80 μm CATR for 10 min on ice. The suspension was solubilized by introduction of up to 4% detergent. Note that in some cases, a 10-fold dilution of the starting membrane suspension was used instead to decrease the sample:detergent ratio. The samples were incubated on ice for 10 min with occasional mixing and centrifuged for 20 min (100,000 × *g*), and the supernatants were recovered. Purified AAC3 was diluted into sample buffer with an appropriate amount of detergent to give 50 μl at the desired final detergent concentration.

For all samples, 5 μl of 50% (w/v) glycerol and enough Coomassie Brilliant Blue G-250 (Serva, Heidelberg, Germany) from a 5% (w/v) stock were added and mixed to give a final detergent/Coomassie ratio of 8 (g/g). 20-μl volumes, equivalent to ∼200 or 20 μg of starting membrane protein or 2–4 μg of purified AAC3, were loaded onto pre-prepared gels. Native high molecular weight markers (GE Healthcare, 17-0445-01) were prepared according to the manufacturer's instructions in the absence of detergent and supplemented 1/10 (v/v) with a 5% (w/v) Coomassie stock suspension. The presence of detergent (1% 12M) did not affect the migration of the marker proteins in control experiments (data not shown). 10 μl was loaded where required.

Electrophoresis was carried out in a cold room (<8 °C) using 5 mA current per gel, and the voltage was limited to 600 V. Gels were run for 1 h using “deep” cathode buffer (50 mm Tricine, 7.5 mm imidazole, 0.02% Coomassie Brilliant Blue G250) and then changed to using “light” cathode buffer (50 mm Tricine, 7.5 mm imidazole, 0.002% Coomassie Blue G250) for a further 2–3.5 h until the dye front started to leach into the anode buffer (25 mm imidazole/HCl, pH 7). The gels were recovered, rinsed, and documented (scanned with a trans-illumination setting) before protein staining with InstantBlue (Novexin, Cambridge, UK) or blotting for antigen detection. SDS-PAGE was carried out in an SE 250 Series Mighty Small II electrophoresis unit using a 12% (w/v) polyacrylamide resolving gel.

##### Western Blotting and Protein Detection

Proteins were transferred to methanol-activated PVDF membranes using a conventional semidry apparatus. Native gels and blotting paper (2 × 3-ply stacks) were soaked in anode buffer for 5 min before transfer for 2 h in a cold room with the current set to 1 mA cm^−2^ gel area and the voltage limited to 25 V. In some cases, native gels were prewashed in cathode buffer with 2% SDS (3 × 200 ml for 1 h) to reduce the interference of Coomassie dye in protein transfer. Membranes were destained with methanol and rinsed in distilled water, and the molecular weight marker bands were visualized and documented using Ponceau S protein stain (Sigma). Stain was removed using 0.1% (w/v) NaOH following the manufacturer's instructions and rinsed in distilled water. SDS gels were transferred using the same semidry apparatus, but for 1.5 h at room temperature using a conventional transfer buffer (25 mm Tris, 192 mm glycine, 20% methanol).

For immuno-detection of antigen, PVDF membranes were incubated at 4 °C overnight in blocking buffer (0.1% Tween 20, 5% (w/v) skimmed milk powder (Marvel) in phosphate-buffered saline) and probed with chicken anti-AAC polyclonal antibody (1:20,000 dilution; AgriSera) or rabbit anti-UCP1 polyclonal antibody (1:5000 dilution; U6382, Sigma) followed by the relevant HRP-conjugated secondary antibody: anti-chicken (1:20,000 dilution; A9046 Sigma) or anti-rabbit (1:10,000 dilution; AP132P Millipore). All antibody incubations were for 1 h at room temperature in blocking buffer. Antigens were visualized on Amersham Biosciences Hyperfilm using an ECL Plus Western blotting detection system (Amersham Biosciences, Little Chalfont, Bucks, UK). A phosphorescent marking pen was used to label the membrane before film exposure so that the film and membrane could be aligned later to accurately assess antigen migration. The apparent molecular mass of each species was estimated by linear interpolation using plots of migration distance *versus* log molecular weight of the protein standards.

##### Size Exclusion Chromatography

Analytical gel filtration was carried out as described previously (27, 39) using a Superdex 200 XK16/60 column (GE Healthcare) equilibrated in buffer (10 mm Tris, pH 7.4, 150 mm NaCl) that included 0–0.4% 12M with or without 0.02% (w/v) Coomassie Blue G-250. All buffers were mixed overnight and filtered before use. In each case, 417 μg of purified AAC3 was supplemented with detergent and Coomassie dye, accordingly, before loading in 1 ml of buffer (flow rate 0.5 ml/min). The column was calibrated with carbonic anhydrase, ovalbumin, conalbumin, aldolase, and ferritin (28-4038-41/28-4038-42, gel filtration calibration kits, GE Healthcare). The protein content of peak fractions was quantified by BCA assay (Thermo Scientific) after treatment with acetone to remove Coomassie dye (see manufacturer's protocol). Coomassie dye was quantified (*A*_610_) after 20-fold dilution into 3% SDS, 10 mm Tris, pH 7.4 (no interference from protein was observed in control experiments).

## RESULTS

### 

#### 

##### Blue Native Gel Electrophoresis of Yeast AAC3

**FIGURE 1.**
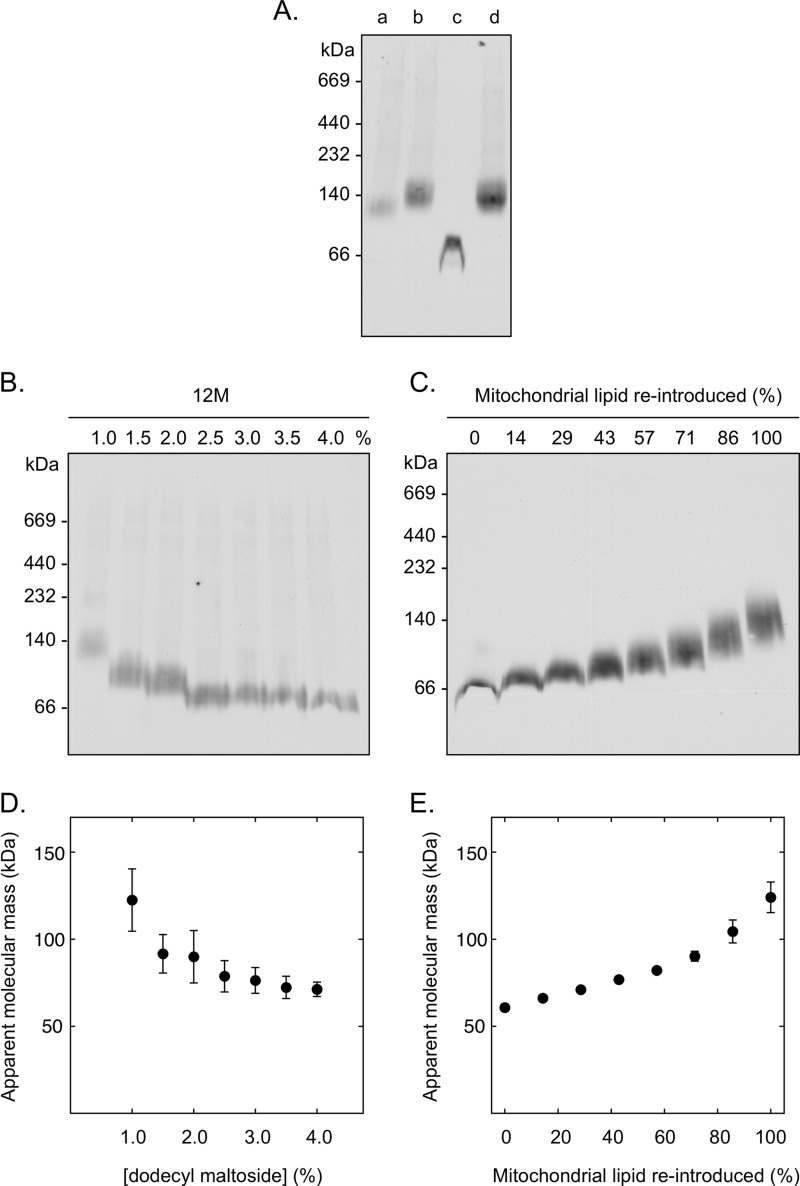
**The influence of detergent concentration and mitochondrial lipid on the apparent molecular mass of yeast AAC3 in blue native gels.** Yeast mitochondrial membranes (200 μg of protein) or purified AAC3 protein (2 μg) were separated in 5–13% (w/v) polyacrylamide gels, and AAC3 was detected by Western analysis. *A*, the apparent molecular mass of AAC3 in 12M-solubilized mitochondrial membranes without (*lane a*) or with pretreatment with 80 μm CATR (*lane b*), when purified (*lane c*) or when purified and combined with the CATR-treated mitochondrial membranes (*lane d*). Accordingly, the antigen present in *lane d* is the sum of the antigen present in *lanes b* and *c*. All samples were prepared in 1% 12M. *B*, the apparent molecular mass of AAC3 in CATR-treated mitochondrial membranes prepared in 1–4% 12M. *C*, the apparent molecular mass of purified AAC3 in 1% 12M with 0–100% of the equivalent amount of mitochondrial lipid present in solubilized membrane samples reintroduced (see “Materials and Methods”). *D* and *E*, the average molecular masses (± S.D.) calculated from repeats of the gels in *B* and *C* (*n* = 3), respectively. The molecular masses (kDa) of protein standards are given to the *left* of each blot.

In mitochondrial membranes solubilized with 12M and separated using common blue native PAGE methods (38), CATR-inhibited AAC3 migrated with an apparent molecular mass of ∼120 kDa when calibrated with soluble marker proteins (Fig. 1*A*, *lane b*, Western blot). This value is within the range of estimates reported for AAC and other mitochondrial carrier proteins on blue native gels (65–120 kDa (15–21, 34)), all of which were interpreted to be protein dimers. In the absence of CATR, AAC3 had a marginally lower molecular mass, but displayed a much weaker signal on Western blots (*lane a*) consistent with a loss in solubility of the protein due to instability. Unless otherwise stated, AAC3 samples were treated with CATR as a standard to keep the protein in a folded state. In contrast to AAC3 in membrane samples, purified AAC3 migrated with a lower apparent molecular mass (∼60 kDa, *lane c*). However, when mixed with solubilized membranes, the purified protein adopted the same high molecular mass observed in the solubilized membrane samples (*cf. lanes d* and *b*). This behavior indicates that a factor present in membrane samples increases the apparent molecular mass of AAC3 and is removed during protein purification. In membrane samples, the apparent mass of AAC3 was sensitive to the concentration of the detergent used during solubilization. The apparent mass of AAC3 decreased from ∼120 to ∼70 kDa over a 4-fold increase in the 12M concentration (Fig. 1, *B* and *D*). This result indicates that the factor that influences the apparent mass of AAC3 can be diluted away by detergent. Importantly, the incremental changes in mass observed were too small to be explained by a change in the oligomeric state of the protein.

##### The Influence of Lipids on the Apparent Size of AAC3

Lipids have been shown to increase the apparent size of the AAC3-detergent micelle in size exclusion studies (31). Following extraction with solvents, the addition of mitochondrial lipids to purified AAC3 increased the mass of the protein in blue native gels too (Fig. 1, *C* and *E*). The apparent mass increased in proportion to the amount of lipid introduced and reached the same mass as observed for AAC3 in solubilized membrane samples (∼120 kDa) at an equivalent lipid concentration. Lipid associated with AAC, therefore, can account for the high mass observed in solubilized mitochondrial samples and may explain some of the variation in values reported for mitochondrial carrier proteins across other studies (15–21, 34).

##### The Effect of Detergent on the Apparent Size of AAC3

**FIGURE 2.**
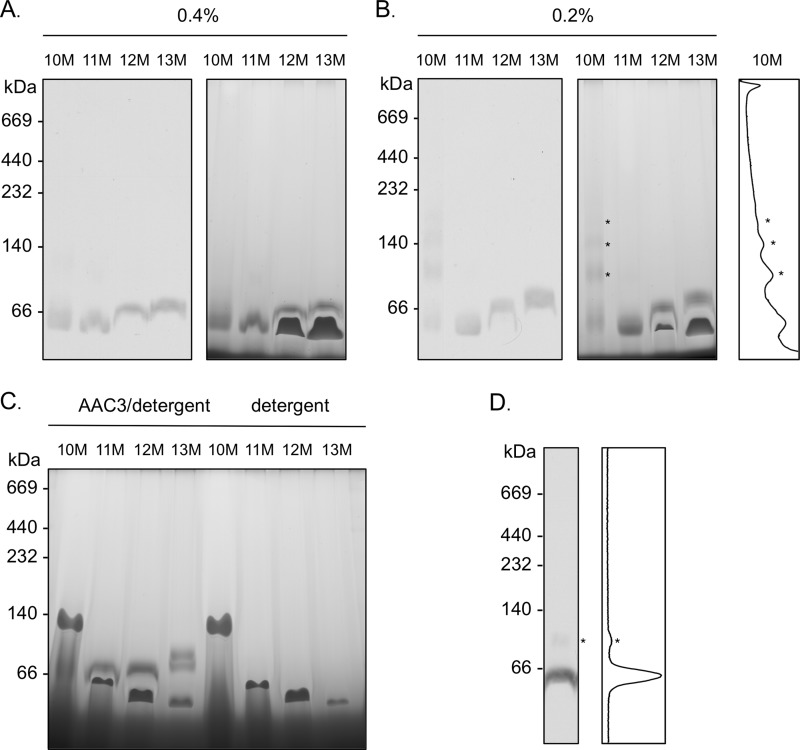
**The influence of free detergent micelles on the migration of purified AAC3 in blue native gels.** Yeast AAC3 was purified in alkyl maltoside detergents of varying micelle size (10–13M; see Ref. 27) and separated in 5–13% (w/v) polyacrylamide gels (4 μg of protein per lane) as described under “Materials and Methods.” *A* and *B*, the migration of purified AAC3 (prepared in detergent as indicated) and protein-free detergent micelles in blue native gels (*right panels*) with the position of AAC3 indicated by Western analysis (*left panels*). The densitometry profile is of *lane 1* (0.2% 10M) of the blue native gel image. *C*, the migration of purified AAC3 and free detergent micelles in samples prepared with detergent at ∼30-fold the critical micelle concentration (2.61% 10M, 0.87% 11M, 0.26% 12M, and 0.05% 13M). *D*, the occurrence of minor higher mass AAC3 species observed occasionally with AAC3 prepared in 0.1–1% 12M (0.5% 12M shown). The molecular masses (kDa) of protein standards are given to the *left* of each gel or blot. Densitometry profiles (ImageJ software) of the relevant lanes are given to clarify the presence of multimeric species (*).

**FIGURE 3.**
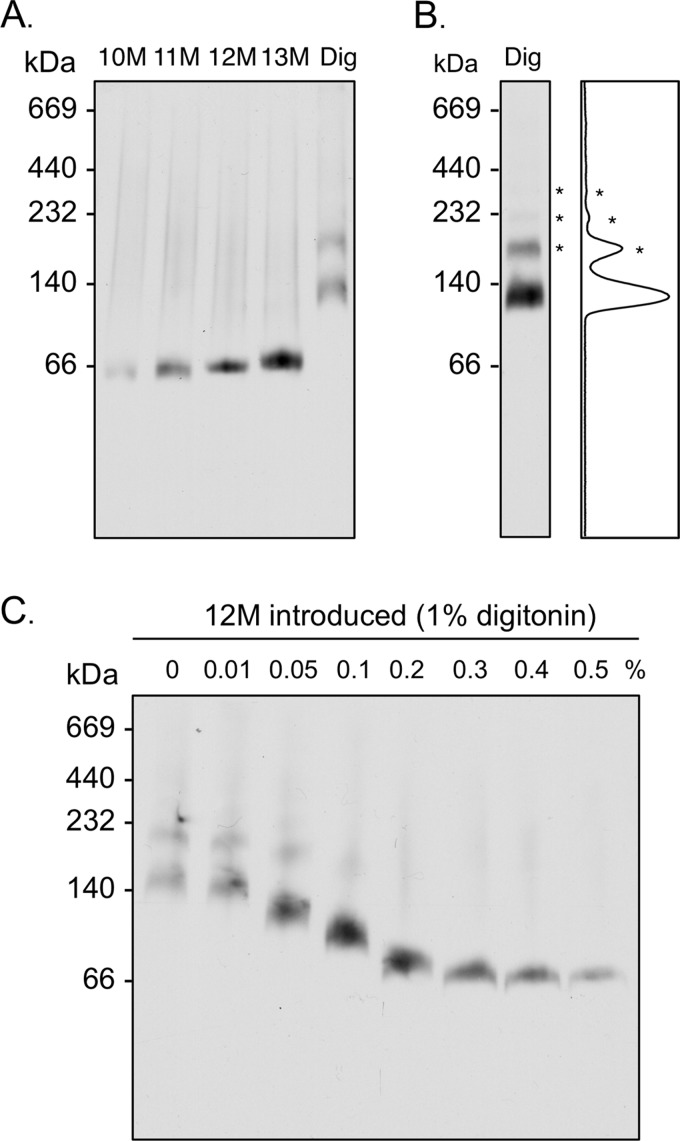
**The apparent molecular mass of AAC3 in alkyl maltoside detergents and digitonin.** Yeast mitochondrial membranes were prepared in detergent with 10-fold less protein present (*cf*. legend for Fig. 1) to minimize the influence of lipids in the protein-detergent micelle. Samples were separated in 6–18% (w/v) polyacrylamide gels, and AAC3 was detected by Western analysis. *A* and *B*, the apparent molecular mass of AAC3 in CATR-treated mitochondrial membranes (20 μg of protein) solubilized with 1% alkyl maltoside detergent (10–13M) or digitonin (*Dig*), as indicated. The occurrence of multimeric AAC3 species (*), observed with mitochondrial membranes solubilized in digitonin, is clarified in *panel B* with a densitometry profile (ImageJ software). *C*, the change in the apparent molecular mass of AAC3 in CATR-treated mitochondrial membranes solubilized in 1% digitonin with the introduction of 0–0.5% 12M. The molecular masses (kDa) of protein standards are given to the *left* of each blot.

Size exclusion studies have demonstrated that purified AAC3 is monomeric in detergent but changes in apparent mass due to the associated detergent micelle (27, 39). In the alkyl maltoside series of detergents, AAC3 is ∼86 kDa in 10M, yet increases up to 134 kDa in 13M as the length of the detergent alkyl chain increases. Here, in the absence of excess lipid, purified AAC3 had an apparent mass of ∼45 to ∼60 kDa in the same detergent series in blue native gels (Fig. 2*A*, *left panel*). In this case, however, the small changes that occurred can be attributed to the interference in migration of AAC3 by free detergent micelles, which also migrated in the gel as a consequence of associated Coomassie dye (Fig. 2*A*, *right panel*). At 30 times the critical micelle concentration, in the absence or presence of AAC3, the position of protein-free detergent micelles was observed for all the detergents tested, as well as the interference that they imparted on the migration of AAC3 (Fig. 2*C*). In each case, the protein was retarded to varying degrees across each lane, giving distorted band profiles. It is worth nothing that similar profiles have been observed for other mitochondrial carrier proteins on blue native gels (*e.g.* Ref. 18). Lowering the detergent concentration did not remove the interference without risking aggregation of the protein (Fig. 2*B*). In some cases, an extra species or even a full array of higher oligomeric states could be seen, depending on the severity of detergent starvation (*e.g.* in 0.2% 10M). On occasion, these species could also be observed with higher detergent concentrations (*e.g.* in 0.5% 12M, Fig. 2*D*), which may be related to the loss of bound detergent during migration of the protein in the gel. However, aggregates were consistently observed in digitonin-solubilized membranes (see below, and Fig. 3, *A–C*).

The overriding effects of excess lipid and detergent could be minimized by using detergent-solubilized membranes at a 10-fold lower protein load than would otherwise be recommended. This approach maximized the dilution of lipids away from the protein by detergent but also appeared to reduce the interference of free detergent micelles in the migration of AAC3 on 6–18% polyacrylamide gels (Fig. 3*A*). Under these conditions, Western detection indicated that AAC3 had an apparent molecular mass of ∼60 kDa in *all* of the alkyl maltoside detergents tested. Coomassie dye, therefore, must have replaced the majority of each alkyl maltoside detergent associated with the protein to give similar mass species. Consistent with these findings, Coomassie dye has been shown to replace virtually all of the 12M associated with the purified lactose transporter LacS (40).

In contrast to the alkyl maltosides, AAC3 exhibited an apparent molecular mass of ∼130 kDa in digitonin (Fig. 3*A*), a detergent commonly used in blue native PAGE studies. Minor species at even higher molecular masses were also detected (Fig. 3, *A* and *B*), consistent with the aggregation of the protein due to limited solubility in this detergent. The 2-fold higher molecular mass of the main AAC3 species in digitonin, however, was not related to a difference in the oligomeric state of the protein. Titration of up to 0.5% 12M into digitonin-solubilized membranes decreased the apparent mass of the species to ∼60 kDa in small increments (Fig. 3*C*). The higher apparent mass, therefore, must have been related to the amount of detergent, lipid, and Coomassie dye associated with the protein, which changed in proportion to the amount of 12M present. AAC3 would appear to retain a relatively large micelle in digitonin that does not exchange fully with Coomassie dye, in contrast to the alkyl maltoside micelles, resulting in a slower migration on gels. This, in addition to the effects of lipid, may explain some of the high molecular masses reported for digitonin-solubilized AAC on blue native gels, which were originally interpreted to be the result of homomeric or heteromeric protein associations (15, 16, 19, 32, 33, 41).

##### The Apparent Size of Mitochondrial Carrier Proteins on Blue Native Gels

**FIGURE 4.**
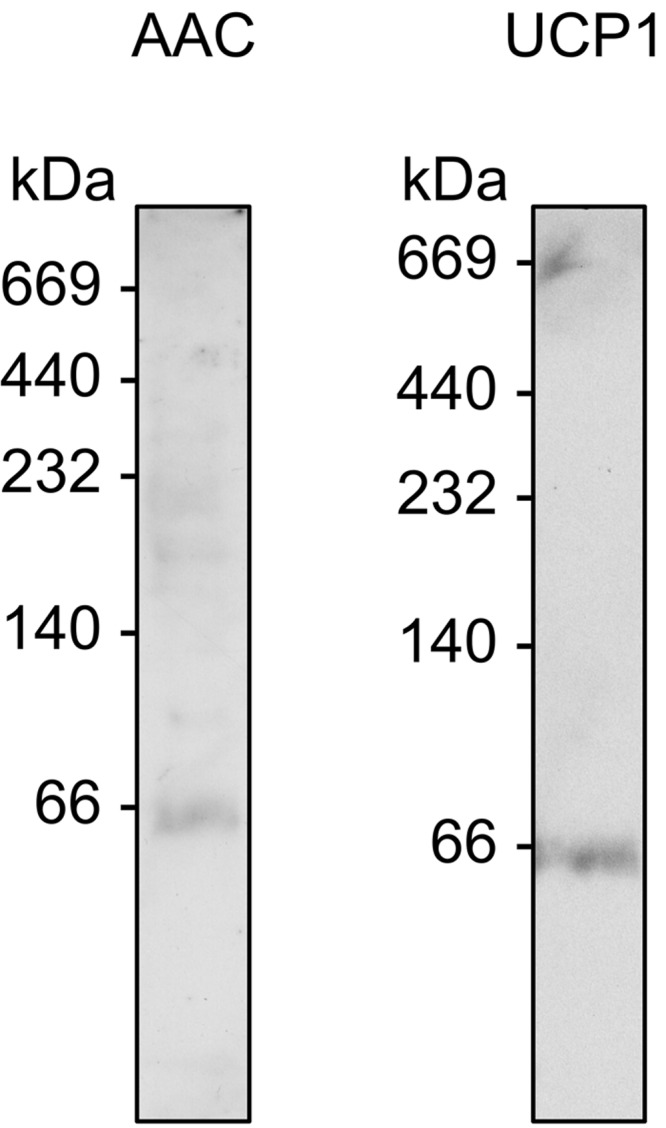
**The apparent molecular mass of native AAC and uncoupling protein-1 in blue native gels.** Mitochondrial membranes from rat liver (AAC) and lamb brown adipose tissue (UCP1) were prepared in 1% 12M and separated in 6–18% (w/v) polyacrylamide gels as described in the legend for Fig. 3. For the detection of AAC3, mitochondrial membranes were pretreated with CATR. 20 μg of protein was loaded per lane. The migration of native AAC and uncoupling protein-1 was immuno-detected by Western analysis (see “Materials and Methods”). The molecular masses (kDa) of protein standards are given to the *left* of each blot.

**FIGURE 5.**
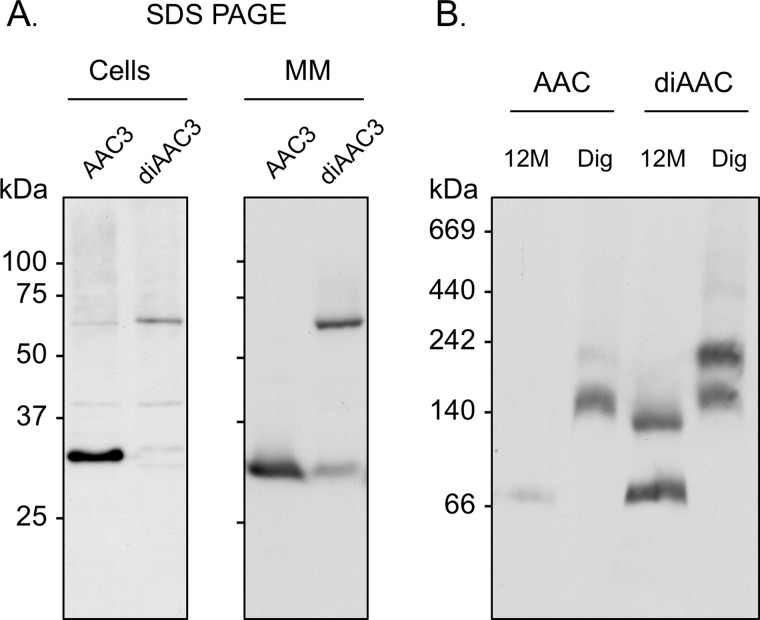
**The calibration of mitochondrial carrier migration with artificial covalently linked AAC dimers.** *A*, whole yeast cells (20 μg of protein) or isolated mitochondrial membranes (*MM*; 7 μg of protein) containing AAC3 or a covalently linked AAC dimer (*diAAC3*) were separated by SDS-PAGE. *B*, CATR-treated mitochondrial membranes (20 μg of protein) containing AAC3 or a covalently linked AAC dimer (*diAAC3*) were separated in 6–18% (w/v) polyacrylamide gels as described in the legend for Fig. 3. AAC3 and diAAC3 were detected by Western analysis. The molecular masses (kDa) of protein standards are given to the *left* of each blot. *Dig*, digitonin.

Using our optimized conditions, mitochondrial carrier proteins from native sources (AAC from liver and UCP1 from brown adipose tissue) migrated with an apparent molecular mass of ∼60 kDa in 12M (Fig. 4), consistent with our findings with the recombinant AAC3 protein from yeast. This would suggest that all of the mitochondrial carrier species tested behave similarly and are in the same oligomeric state in blue native gels. Whether these species represent monomers or dimers, however, is not immediately clear as membrane proteins have been shown to bind more Coomassie dye than the soluble marker proteins (g/g protein) and so do not migrate at their expected molecular mass (40). To act as a positive control for dimers, we generated covalently linked tandem dimers of AAC3 (diAAC3; see “Materials and Methods”), as has been done for yeast AAC2 (35). Expression of the construct in an AAC-deficient strain of yeast allowed growth on glycerol, a nonfermentable carbon source, indicating that diAAC3 is functional and can support oxidative phosphorylation. When separated on denaturing SDS gels, diAAC3 was ∼64 kDa in whole cells, as expected, but was 64 and 32 kDa in mitochondrial membrane samples, indicating that diAAC3 was in part proteolytically cleaved during membrane isolation (Fig. 5*A*). When these membranes were solubilized in 12M and separated on blue native gels, AAC species with apparent molecular masses of ∼130 and ∼60 kDa were observed, corresponding to the intact and cleaved proteins, respectively (Fig. 5*B*). Similarly, when digitonin was used as the detergent, again two species were observed but at higher mass values (∼230 and ∼140 kDa). Importantly, in both cases, it was the lower, cleaved species rather than the covalent dimer that matched the migration of conventional AAC3, indicating that AAC3 and the other mitochondrial carrier proteins tested migrate as monomers in blue native gels.

When separated according to charge/mass by blue native PAGE, AAC3 appeared smaller than when separated by size alone with size exclusion chromatography (∼60 kDa when compared with ∼115 kDa in 12M (27, 39)). Less Coomassie dye may be required to replace the detergent bound to AAC3 (g/g protein) or, alternatively, a relatively high amount of Coomassie dye may bind to AAC3, giving the protein a higher net charge and mobility on gels than expected. To distinguish between these possibilities, we assessed AAC3 under blue native gel-like conditions by using size exclusion chromatography. In blue native PAGE, proteins are typically exposed to 0.02% Coomassie dye in the cathode buffer, but are exposed to higher concentrations in the sample buffer and during electrophoresis as the Coomassie dye forms a concentrated running front. Also, as proteins migrate on gels, the local detergent concentration depletes progressively as there is no detergent cast in the gel, and free detergent, in equilibrium with the Coomassie-detergent micelles, will not migrate with the protein. Therefore, as proteins migrate, they will be exposed to an increasing Coomassie dye:detergent ratio. To mimic these conditions in size exclusion experiments, we introduced 0.02% Coomassie dye to both purified AAC3 samples and the chromatography buffer and systematically decreased the 12M concentration present.

**FIGURE 6.**
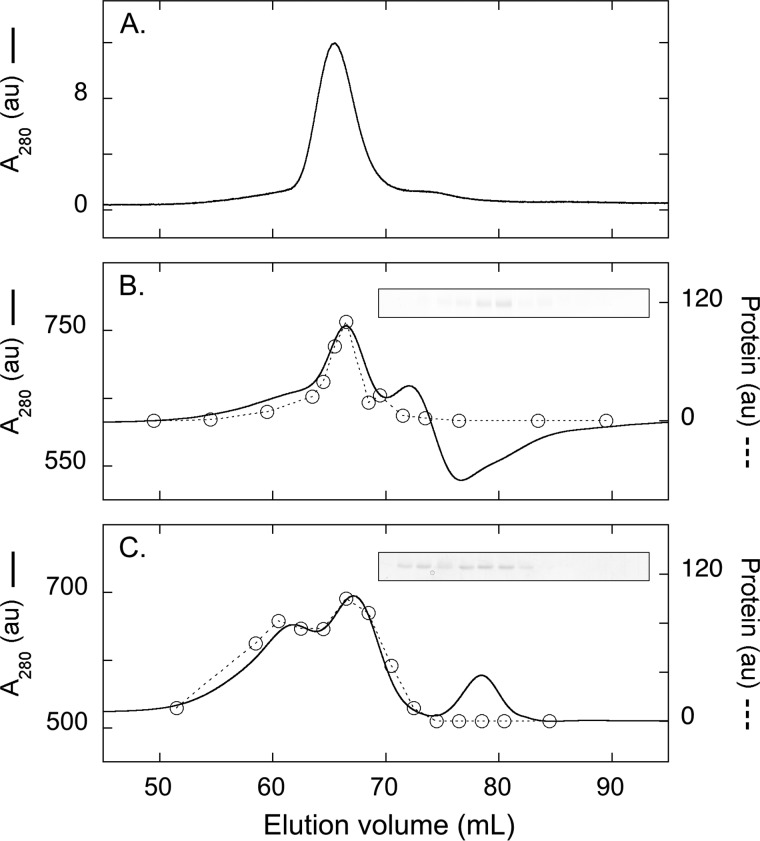
**Size exclusion chromatography of purified AAC3 under blue native gel-like conditions.** *A–C,* elution profiles of purified AAC3 in 0.1% 12M (peak = 144 kDa) (*A*), 0.1% 12M with 0.02% Coomassie dye (peak = 133 kDa) (*B*), or 0.004% 12M with 0.02% Coomassie dye (peak 1 = 125 kDa and peak 2 = 185 kDa) (*C*). AAC3 was loaded in 0.1% 12M sample buffer supplemented with (*B* and *C*) or without (*A*) 0.02% Coomassie dye. The protein profile (*dashed line*) was quantified by densitometry of the eluted fractions on Coomassie-stained SDS gels (*inset figures*). See Table 1 and Fig. 7 for species composition. The column was calibrated with molecular mass standards (see “Materials and Methods”). *au*, arbitrary units.

**FIGURE 7.**
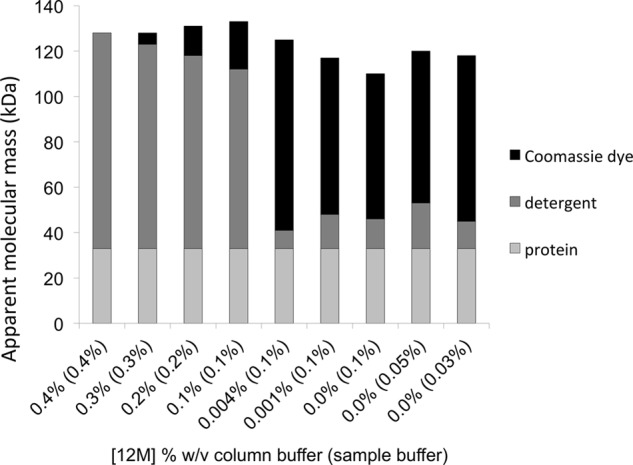
**The size and composition of AAC3 species under blue native gel-like conditions.** Apparent molecular masses were estimated by size exclusion chromatography in the presence of 0.02% Coomassie dye and the indicated amount of 12M detergent. See Table 1 for details.

In 0.1% 12M, purified AAC3 eluted with an apparent molecular mass of 144 kDa (Fig. 6*A*). This value is higher than past estimates (27), which relates to changes in the protein composition of the commercial calibration kit used (see “Materials and Methods”). In the presence of 0.02% Coomassie dye, a high background signal was observed due to the strong absorbance of the dye at 280 nm. Even so, a clear peak corresponding to AAC3 could be observed that eluted with an apparent molecular mass of 133 kDa (see protein profile of the eluted fractions, Fig. 6*B*). When the 12M concentration in the chromatography buffer was decreased to below the critical micelle concentration, most of the AAC3 present showed a small decrease in apparent molecular mass (to between 111 and 125 kDa, Fig. 6*C* and Table 1), whereas a minor fraction showed an increase (to ∼180 kDa) consistent with oligomerization due to limited solubility, as observed in some conditions in blue native gels (*e.g.* Fig. 2*D*). A third peak observed corresponded to the elution of protein-free Coomassie-detergent micelles. Under these conditions, quantification of the protein and Coomassie dye in the major peak fractions indicated that up to 2.5 g of Coomassie dye per g of protein associated with AAC3 despite relatively little change in the apparent size of the species (Table 1). Estimation of the composition revealed that most of the protein-bound detergent must have exchanged for Coomassie dye (Table 1 and Fig. 7), similar to the situation observed on blue native gels with different alkyl maltoside detergents (Fig. 3*A*). These data are only compatible with a model that considers the predominant AAC3 species to be monomeric, in line with our previous studies (27, 39). AAC3 would appear to bind over 2.5-fold more Coomassie dye than other membrane proteins (*e.g.* 0.35–0.59 g/g of protein for respiratory complexes I, III, IV, and V (42) or 0.8 g/g of protein for LacS (40)). The smaller apparent mass of AAC3 determined by blue native PAGE when compared with size exclusion chromatography, therefore, most likely reflects a faster than expected migration rate in gels due to a higher than expected associated charge, rather than a real difference in mass.

**TABLE 1:** **The size and composition of AAC3 species under blue native gel-like conditions** Apparent molecular masses were estimated by size exclusion chromatography in the presence of 0.02% Coomassie dye and the indicated amount of 12M detergent. See Fig. 6 for examples. Protein and Coomassie dye in the major peak fractions were quantified as described under “Materials and Methods.”

[Dodecyl-β-d-maltoside]		Apparent molecular mass	Coomassie:protein	Species composition		
Column buffer (w/v)	Sample buffer (w/v)			Protein*^a^*	Coomassie*^b^*	Detergent*^c^*
%	%	*kDa*	*g/g*	*kDa*		
0.4	0.4	128	0.0	33	0	95
0.3	0.3	128	0.1	33	5	90
0.2	0.2	132	0.4	33	13	85
0.1	0.1	133	0.6	33	21	79
0.004	0.1	125	2.5	33	84	8
0.001	0.1	117	2.1	33	69	15
0.000	0.1	111	1.9	33	64	13
0.000	0.05	120	2.0	33	67	20
0.000	0.03	118	2.2	33	73	12

*^a^* Calculated from the amino acid composition of yeast AAC3.

*^b^* Calculated from the protein mass multiplied by the Coomassie:protein ratio.

*^c^* Calculated by subtracting the apparent masses of the protein and Coomassie dye from the total apparent molecular mass of the species. This portion may represent some lipid as well.

## DISCUSSION

Blue native PAGE is a popular and convenient technique to separate and study proteins under nondenaturing conditions and has been used extensively to report the oligomeric state of mitochondrial carrier proteins (15–21, 34). In all studies to date, mitochondrial carriers have been reported to be dimeric, migrating with an apparent molecular mass of between 65 and 120 kDa. Here, we have scrutinized the behavior of the mitochondrial carrier protein AAC3 on blue native gels. We find that the apparent mass of the protein is strongly influenced by the lipid and detergent present in a manner that is unrelated to changes in oligomeric state. When these effects are minimalized, AAC3 and other mitochondrial carrier proteins migrate as ∼60-kDa species. Assessment by size exclusion chromatography suggests that purified AAC3 is larger than this on blue native gels but migrates erroneously due to an unexpectedly high amount of bound Coomassie dye. Importantly, calibration of gels with an artificial dimer construct indicates that the 60-kDa species observed for AAC3 and other mitochondrial carrier proteins is monomeric.

The migration of mitochondrial carrier proteins on blue native gels is complicated by several factors. We found that the presence of endogenous lipids, and the degree to which they are diluted by detergent, had a major effect of the apparent molecular mass of AAC3. Consistent with this, others have observed that yeast Aac2p in digitonin-solubilized mitochondria is ∼109 kDa from wild type yeast yet is only 94 kDa in mitochondria from a cardiolipin-deficient strain (Δ*crd1*), where the mitochondrial lipid content is lower (19). These trends are also in agreement with our previous size exclusion experiments where the introduction of lipids to AAC3 purified in 12M substantially increased the Stokes radius of the protein-detergent micelle (31). As well as increasing the effective size, lipids may also compete with, and limit, the binding of Coomassie dye, which could lead to further retardation of the protein on gels. This may explain the particularly high increase in the apparent mass of AAC3 observed in the presence of lipid (Fig. 1).

The concentration and type of detergent present also have a major influence on the behavior of AAC3 in blue native gels. Heuberger *et al.* (40) concluded that virtually all of the 12M associated with the membrane protein LacS is replaced by Coomassie dye. Similarly, for the various alkyl maltosides, we find that much of the detergent associated with AAC3 is likely to have been replaced (Fig. 3*A*), albeit not all of it. Size exclusion experiments indicate that a small portion of 12M is retained even at high Coomassie dye:detergent ratios (Fig. 7), whereas the difference in the susceptibility of AAC3 to aggregate in each of the alkyl maltoside detergents (Fig. 2*B*) would also suggest that at least some detergent must remain bound to the protein. We found that protein-free alkyl maltoside detergent micelles are able to migrate in gels as distinct Coomassie-bound species. Although the exact composition of the species is not known, the behavior on gels is counterintuitive: detergents that form larger micelles appear to migrate faster than detergents that form smaller ones. It is possible that a smaller micelle size may limit the amount of Coomassie dye that can be taken up and therefore the amount of charge that is needed for migration. As a consequence of the free detergent micelle migration, the progress of AAC3 is hindered to varying degrees (Fig. 2), leading to erroneous trends in the apparent mass of the protein and “inverted smile”-shaped bands. This interference is reduced with low amounts of solubilized membrane samples loaded onto 6–18% polyacrylamide gels, revealing a similar mass of AAC3 in all the alkyl maltoside detergents tested. The improvement may relate to the altered acrylamide concentrations used or to the loss of defined detergent micelles, dispersed by the extra lipids and protein present in membrane samples. Alternatively, it may relate to a tighter band associated with a lower AAC3 load that overlaps less with the migration of the free detergent micelles.

AAC3 appeared ∼2-fold larger in blue native gels with mitochondrial membranes solubilized in digitonin when compared with 12M, yet is monomeric in both conditions. The extra apparent mass in digitonin, therefore, must relate to the larger micelle that forms around AAC with this detergent, as observed in size exclusion studies where monomeric AAC3 was estimated to be 115 kDa in 12M but larger than 180 kDa in digitonin (27). This suggests that relatively little of the digitonin bound to AAC3 is lost in exchange for Coomassie dye, unlike the situation with the alkyl maltoside detergents. Membrane proteins, in general, are reported to be larger in blue native gels when prepared in digitonin compared with 12M (34), consistent with a digitonin forming a larger micelle, although the presence of more lipids associated with digitonin-solubilized proteins could also explain the apparent increase in size (34).

Heuberger *et al.* (40) have demonstrated that the apparent mass of several membrane proteins determined by blue native PAGE requires a correction factor of 1.8 to account for the extra Coomassie dye that binds relative to soluble marker proteins (*i.e.* the transporters are ∼1.8-fold smaller than they appear on gels). In agreement with this, LacS purified in 12M and exchanged into Coomassie dye was found to bind 0.8 g of Coomassie dye per g of protein. Other small membrane proteins also appear to obey this relationship (*e.g.* rhodopsin (43), the carnitine transporter CaiT (44), the urea transporter ApUT (45), the glutamate transporter GltP_Ec_ (46), and a truncated version of the copper transporter CopB (47)). Applied here, the correction factor would predict that AAC3 is ∼33 kDa (∼60 kDa), consistent with our observations that AAC3 is monomeric in blue native gels. In size exclusion experiments, however, we found that purified AAC3 was able to bind much more Coomassie dye (up to 2.5 g/g of protein) than this correction factor would otherwise suggest, giving a final complex of ∼120 kDa. This relatively high degree of binding most likely relates to the small size and disproportionately large hydrophobic surface of AAC3 when compared with the other membrane proteins studied (40, 42). Importantly, the considerable charge associated with the bound Coomassie dye means that the complex is also likely to migrate faster on gels and so appear smaller. This may explain why the AAC3 species is only ∼60 kDa on gels and a correction factor larger than 1.8 is not required.

Mitochondrial carriers are inherently unstable. We found that CATR was required to stabilize AAC3 on blue native gels. Most mitochondrial carriers, however, cannot be stabilized in this way, and so to what extent their migration on blue native gels reflects a native conformation is not clear. Even in the presence of CATR, small amounts of AAC3 can aggregate into higher molecular mass species under particular conditions. Protein “laddering” has been observed with other membrane proteins (44, 45, 47, 48) and is consistent with the occurrence of less favorable solubilization conditions. This typically occurs when the detergent concentration is limiting but can occur at higher concentrations also with particular detergents (*e.g.* in digitonin or, on occasion, in 12M). In general, there is a good correlation between membrane protein profiles observed on blue native gels (taking into account correction factors) with those observed using other sizing techniques, suggesting that the real state of the protein in detergent is reported despite the presence of Coomassie dye (47, 48). Of note, minor AAC multimers were identified by analytical ultracentrifugation in preparations of bovine AAC1 in Triton X-100, where the protein was also found to be predominantly monomeric (30).

Mitochondrial carrier proteins were originally thought to be dimeric in both form and function. However, there is now considerable evidence from various techniques, as well as retrospective analysis of past data (reviewed in Ref. 31), to show that mitochondrial carriers are in fact monomeric. Here, we have addressed the behavior of mitochondrial carrier proteins in blue native PAGE, a technique that has provided support for the presence of dimers in the past. Our observations are consistent with previous work but show clearly that the carrier species in question are monomeric not dimeric.

## Footnotes

2 The abbreviations used are:

CATR: carboxyatractyloside
Coomassie dye: Coomassie Blue G-250
10M: decyl-β-d-maltoside
11M: undecyl-β-d-maltoside
12M: dodecyl-β-d-maltoside
13M: tridecyl-β-d-maltoside
AAC: ADP/ATP carrier
diAAC3: covalently linked AAC dimer
Tricine: *N*-[2-hydroxy-1,1-bis(hydroxymethyl)ethyl]glycine
UCP1: uncoupling protein-1.
